# Nanopillars with E-field accessible multi-state (N ≥ 4) magnetization having giant magnetization changes in self-assembled BiFeO_3_-CoFe_2_O_4_/Pb(Mg_1/3_Nb_2/3_)-38at%PbTiO_3_ heterostructures

**DOI:** 10.1038/s41598-018-19673-8

**Published:** 2018-01-26

**Authors:** Xiao Tang, Ravindranath Viswan, Min Gao, Chung Ming Leung, Carlos Folger, Haosu Luo, Brandon Howe, Jiefang Li, Dwight Viehland

**Affiliations:** 1 Department of Materials Science and Engineering, Virginia Tech, Blacksburg, VA 24060 USA; 2 INTER Materials LLC, Pulaski, VA 24301 USA; 3 Ceramic Engineering Department, Corning Inc., Corning, NY 14831 USA; 4 State Key Laboratory of High-Performance Ceramics and Superfine Microstructure, Shanghai Institute of Ceramics, Chinese Academy of Sciences, Shanghai, 201800 China; 5 Materials and Manufacturing Directorate, Air Force Research Laboratory, Wright-Patterson Air Force Base, Dayton, Ohio, 45433 USA

## Abstract

We have deposited self-assembled BiFeO_3_-CoFe_2_O_4_ (BFO-CFO) thin films on (100)-oriented SrRuO_3_-buffered Pb(Mg_1/3_Nb_2/3_)_0.62_Ti_0.38_O_3_ (PMN-38PT) single crystal substrates. These heterostructures were used for the study of real-time changes in the magnetization with applied DC electric field (E_*DC*_). With increasing E_*DC*_, a giant magnetization change was observed along the out-of-plane (easy) axis. The induced magnetization changes of the CFO nanopillars in the BFO/CFO layer were about ΔM/M_*rDC*_ = 93% at E_*DC*_ = −3 *kv*/*cm*. A giant converse magnetoelectric (CME) coefficient of 1.3 × 10^−7^ s/m was estimated from the data. By changing E_*DC*_, we found multiple(N ≥ 4) unique possible values of a stable magnetization with memory on the removal of the field.

## Introduction

Multiferroic materials have multiple ferroic order parameters, such as polarization and magnetization^1–3^. Because of the co-existing ferroic properties, these materials are capable of modulating magnetism by electric field via a converse magnetoelectric (ME) effect^4^. The ME effect offers possible advantages, such as: low power consumption (passive)^2^, fast response times^5^ and multiple magnetic states^6^. Because of these unique characteristics, multiferroic materials have been studied for memory devices, such as electric-field-controlled magnetic random access memory^1^. The magnetoelectricity in the best single phase (natural) materials, such as BiFeO_3_ (BFO), is small^7–9^; however, artificial heterostructures consisting of multiple ferroic layers (magnetostrictive and piezoelectric) possess dramatically higher ME couplings^10–12^. There are several types of heterostructure that have been studied to enhance the ME coupling^3,13^, such as multi-layer (noted as 2–2), particles in a matrix (noted as 0–3) and vertically integrated nanopillars (noted as 1–3). However, (2–2) and (0–3) epitaxial heterostructures on substrates suffer from clamping effects and high leakage currents^14^. Accordingly, the (1–3) heterostructure offers much promise because of high magnetic anisotropy, and significantly reduced clamping.

Cobalt ferrite (CoFe_2_O_4_, CFO) is a well-known magnetostrictive material with a large magnetic anisotropy^15–17^. Nanopillar heterostructures of BFO-CFO have been epitaxially deposited on SrTiO_3_ (STO)^18–21^. STO substrates have close lattice parameter matching with both phases of the BFO-CFO layer, and thus there is an intimate lattice contact in the (1–3) heterostructure^22^. This intimate lattice contact transfers E-field induced strain in the piezoelectric phase to the magnetostrictive one, resulting in induced magnetization changes in the nanopillars. As a consequence, (1–3) heterostructures possess significantly larger ME coefficients compared with single phase ME materials^23^. Furthermore, lateral strain control is limited beyond a critical thickness (∼10 0nm), above which the strain may fully relax^23^: (1–3) heterostructures thus can have significantly reduced thickness effects, resulting in thicker films with higher ME coupling. Wang *et al*.^15^ showed that by controlling the size, shape and volume fraction ratio of the CFO nanopillar phase, the magnetic properties could be tailored. The nanopillar morphology was shown to provide a contribution to the shape anisotropy^15^ that can constrain the rotation of the magnetization direction. This may offer an approach to a new multi-state magnetization dependent on electric field history.

Because the STO substrate lacks a piezoelectric effect, the piezoelectric response of nanopillar heterostructures of BFO-CFO is completely determined by the BFO matrix phase of the epitaxial layer. In addition, BFO-CFO heterostructures suffer from notable leakage current^21,24^, as the E-field must be applied to the BFO-CFO layer to induce piezoelectric shape changes^18,20,21^. This limits the BFO-CFO/STO heterostructures from realizing the potential of its full ME coefficients^21^. Recently, a CFO single phase layer was deposited on piezoelectric Pb(Mg_1/3_Nb_2/3_)_0.62_Ti_0.38_O_3_ (PMN-38PT) substrates by Wang *et al*.^16,25,26^. Because of the high piezoelectric coefficient of PMN-38PT, a large magnetization change was observed in the CFO film under application of a large E-field. Furthermore, the E-field was applied to the PMN-PT substrate rather than the CFO layer, resulting in reduced leakage currents, and enhanced magnetization changes in the CFO layer.

A vertically integrated nanopillar BFO-CFO heterostructure has also recently been epitaxially deposited on SrRuO_3_ buffered Pb(Mg_1/3_Nb_2/3_)_0.70_Ti_0.30_O_3_ (SRO/PMN-30PT) substrates^27^. This vertically integrated (1–3) heterostructure allows for a large magnetic anisotropy, which enables E-field tunable magnetic switching^28^. As a substrate, PMN-38PT has a small lattice mismatch with both CFO and BFO^17,29^. Unlike BFO-CFO/STO heterostructures, the E-field induced strain of BFO-CFO/PMN-PT heterostructures is mainly provided by domain reorientation in the PMN-PT substrate^28,30,31^. The combination of the large d_33_ value of PMN-38PT^28^ and the unique constraint of the vertically integrated two-phase structure results in a large ME coefficient. Recently, a giant ME coupling has been reported for BFO-CFO/PMN-30PT heterostructures by our group^28^. However, the number of magnetization states under different E-fields was not studied. Here, we report a self-assembled two-phase vertically integrated BFO-CFO/SrRuO_3_/PMN-38PT heterostructure by pulsed laser deposition (PLD). This BFO-CFO heterostructure possesses large magnetization changes in the CFO nanopillars by application of a DC electrical bias (*E*_*DC*_) to the substrate. A giant ME coefficient has been obtained. It was also found feasible to access multiple (${\rm{N}}\ge 4$) stable magnetization states with memory.

## Results and Discussion

**Figure 1:**
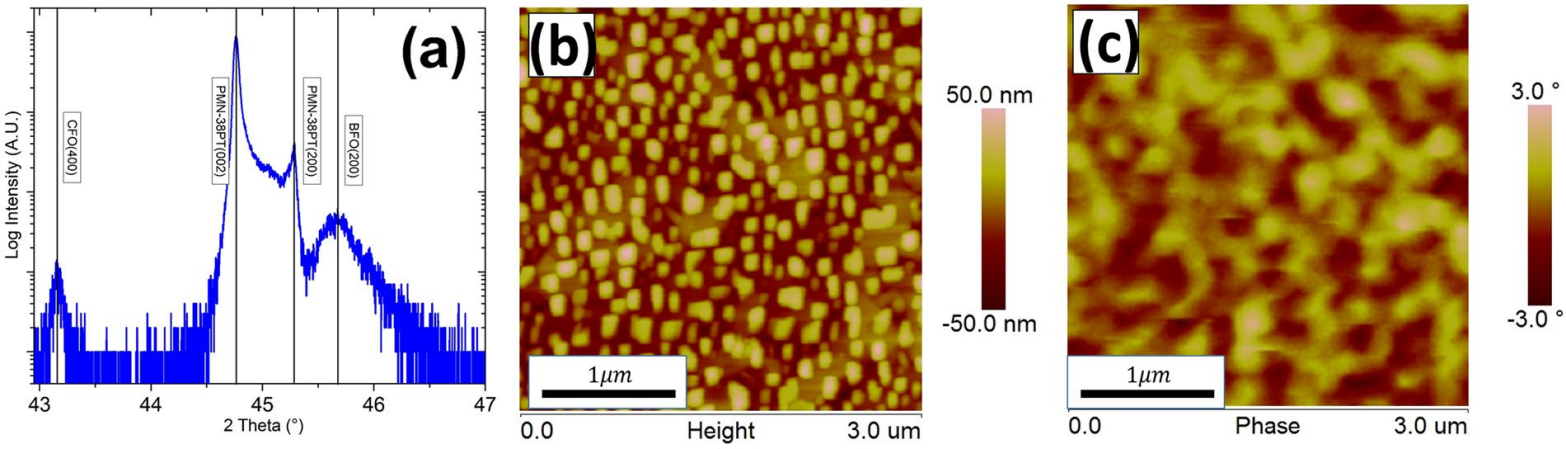
For BFO-CFO/SRO/PMN-38PT heterostructure, (**a**) 2θ scan showing an epitaxial growth BFO-CFO heterostructure, (**b**) AFM image shows square nanopillars embedded in a matrix, and (**c**) MFM phase image indicating magnetic domain orientations.

Figure 1(a) shows an XRD line scan for a BFO-CFO/SRO/PMN-38PT heterostructure. The figure illustrates that the stable phase of PMN-38PT substrates has a T structure with d-spacings of ${a}_{T}$ = 4.046 Å and ${c}_{T}$ = 4.002 Å. The results also show that the BFO-CFO nanocomposite layer grows epitaxially on PMN-38PT. The 2 ${\rm{\theta }}$ value for the (400) peak of CFO was 43.17° and that for the (200) peak of BFO was 45.71°, corresponding to d-spacings of 8.376 Å and 3.967 Å respectively. Compared with bulk BFO (3.960 Å^32^) and CFO (8.392 Å^33^), CFO undergoes a −0.179% strain (compressive) and BFO undergoes a 1.640% strain (tensile) along the OP direction. Figures 1(b,c) show AFM and MFM images for a BFO-CFO/SRO/PMN-38PT heterostructure. Part (b) shows an AFM image evidencing that a self-assembled square-like nanopillar morphology embedded in a matrix formed; and Part (c) shows a MFM image of the phase signal, demonstrating that different nanopillars have dark and light contrasts, indicating that the magnetic domain orientations do not have a preferred distribution amongst equivalent directions.

**Figure 2:**
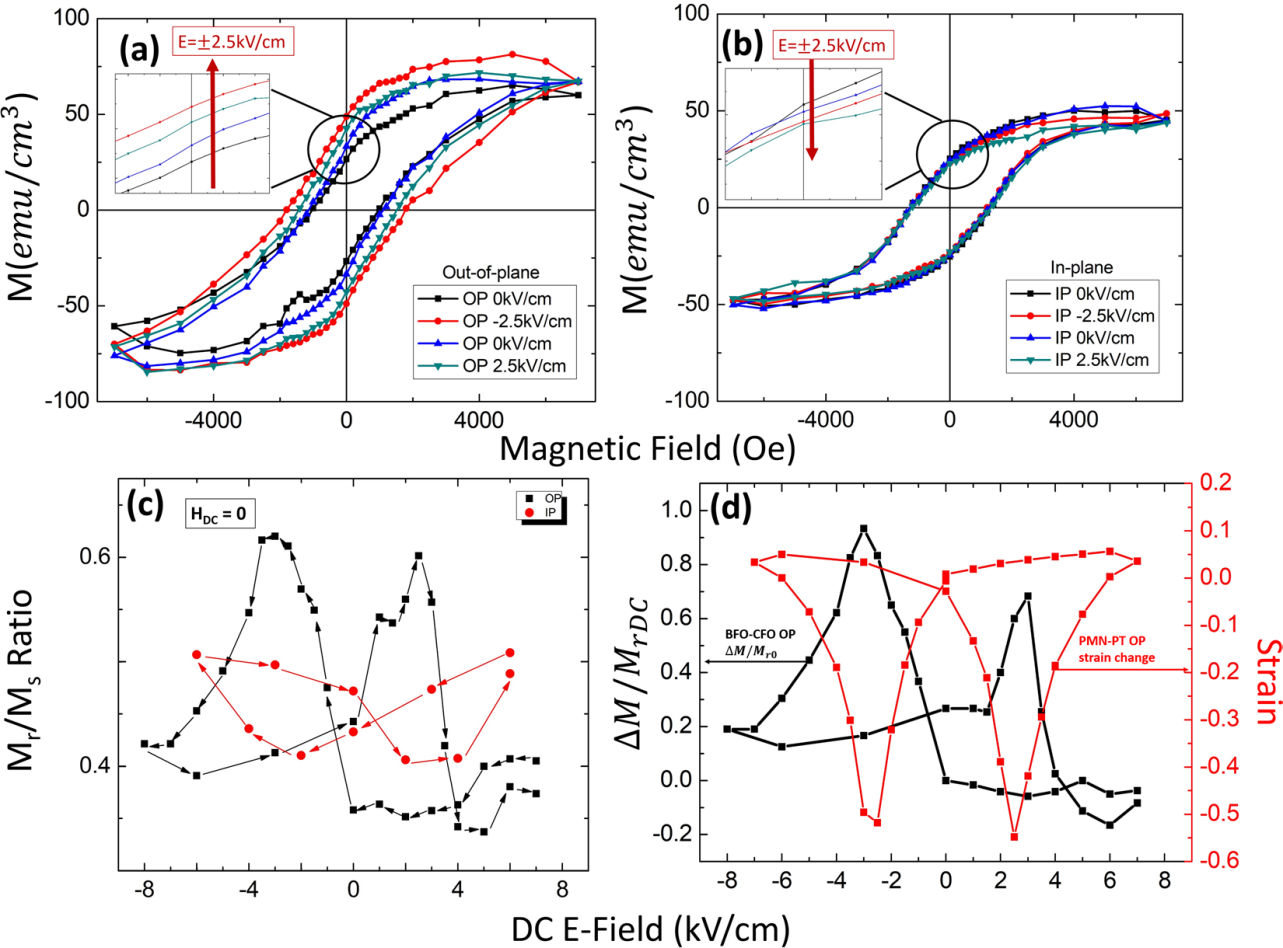
Magnetization measurements for BFO-CFO/SRO/PMN-38PT. (**a**) M-H loop under different E_*DC*_, out-of-plane. (**b**) M-H loop under different E_*DC*_, in-plane. (**c**) ${M}_{r}/{M}_{s}$ ratio with changing E_*DC*_, both out-of-plane and in-plane directions. (**d**) PMN-38PT substrate strain change with E_*DC*_ (right axis), and ${\rm{\Delta }}M/{M}_{ro}$ (left axis) of BFO-CFO/SRO/PMN-38PT as a function of E_*DC*_. The DC electric field E_*DC*_ was applied out-of-plane in all measurements.

Magnetization measurements were then performed in response to an *E*_*DC*_ applied to the substrate. The electric field was applied along the out-of-plane direction. Figure 2(a,b) show the M-H loops under various *E*_*DC*_ along the OP and IP directions, respectively. From these data, it can be clearly seen that the easy axis of the CFO nanopillars lies along the OP direction. This is a reflection of the shape anisotropy of the nanopillar structure, which has a much larger thickness than width. As shown in the insert of Fig. 2(a,b), the remnant magnetization (${M}_{r}$) increases along OP with increasing E_*DC*_. Furthermore, ${M}_{r}$ decreases with increasing *E*_*DC*_ along IP, although the change is small. This is due to the combination of the anisotropy of the magnetostriction coefficient of CFO (${\lambda }_{CFO}$)^16^ and the piezoelectric coefficient of PMN-xPT. The E-field induced strain in BFO-CFO/PMN-PT heterostructure is mainly due to the domain reorientation in the PMN-PT substrate, unlike that in BFO-CFO/STO heterostructures^28,30,31^. The BFO matrix has an important effect in imparting a large shape anisotropy to the CFO nanopillars. With increase of the aspect ratio of the CFO nanopillars, the shape anisotropy is significantly enhanced^15^, altering the easy axis from IP to OP directions. From the right hand axis of Fig. 2(c), it can be seen that the PMN-38PT substrate undergoes a compressive stress along the OP direction under *E*_*DC*_, resulting in the BFO-CFO nanocomposite layer experiencing a tensile stress along IP. Since *λ*_*CFO*_ < 0, under a tensile IP stress, the easy axis of the CFO will rotate towards the OP direction^34^. As a consequence, the nanopillars will experience an increase in ${M}_{r}$ with increasing *E*_*DC*_, and vice versa a lower ${M}_{r}$ along the IP.

The remnant-to-saturation (${M}_{r}/{M}_{s}$) magnetization ratio in response to *E*_*DC*_(−7 kV/cm < *E*_*DC*_ < 7 kV/cm) applied to the PMN-38PT substrate is shown in Fig. 2(c). Data are given for E_DC_ applied along the OP and IP directions. As can be seen in Fig. 2(c), the ${M}_{r}/{M}_{s}$ ratio as a function of E_DC_ exhibited a clear butterfly-like shape, similar, but inverted to the ${\rm{\varepsilon }}-{\rm{E}}$ response of the PMN-38PT substrate. The highest values of the ${M}_{r}/{M}_{s}$ ratio were 0.62 under $-3kV/cm$ and 0.60 under $2.5kV/cm$. As shown in Fig. 2(c), after ${{\rm{E}}}_{DC}$ reached $-7\,{\rm{kV}}/{\rm{cm}}$, the ${M}_{r}/{M}_{s}$ ratio approached a stable plateau at a value of $\sim 0.40$. Upon removal of E_*DC*_, the value of ${M}_{r}/{M}_{s} \sim 0.4$ was maintained. Similarly, after E_*DC*_ reached $+7{\rm{kV}}/{\rm{cm}}$, ${M}_{r}/{M}_{s}$ approached a stable plateau at a value of ~0.35. The IP direction had an opposite trend compared to the OP, exhibiting smaller changes in the ${M}_{r}/{M}_{s}$ ratio with increasing E_*DC*_ that were inverted with respect to the OP data. This inversion occurs because the easy magnetization axis is aligned with the OP direction, imparting to the IP axis relatively lower values of both ${M}_{r}$ and coercivity.

Next, ${\rm{\Delta }}M={M}_{rDC}-{M}_{r+o}$ (difference between ${M}_{r}$ under ${{\rm{E}}}_{DC}\ne 0$ and ${M}_{r}$ under ${{\rm{E}}}_{DC}=0$ after positive E_*DC*_) was calculated. To normalize the change of ${\rm{\Delta }}M$, the value of ${\rm{\Delta }}M/{{\rm{M}}}_{rDC}$ verses E_*DC*_ was calculated as shown in Fig. 2(d) on the left axis. ${\rm{\Delta }}M/{{\rm{M}}}_{rDC}$ verses E_*DC*_ applied along the OP direction had the same inverted butterfly-like shape as the data for the ${M}_{r}/{M}_{s}$ ratio. Also, ${\rm{\Delta }}M/{{\rm{M}}}_{rDC}$ as a function of E_*DC*_ had similar values above the electric coercive field of PMN-38PT (see right axis in Fig. 2(d)), which were slightly greater than zero. The highest value of ΔM/M_*DC*_ (∼0.90) was found near E_*DC*_ = −3 kV/cm, which corresponded to the electric coercive field under negative polarity. This evidences that BFO-CFO/SRO/PMN-38PT heterostructures have their largest E_*DC*_ induced ME coupling when the polarization reverses. The maximum value of ${\rm{\Delta }}M/{{\rm{M}}}_{rDC}$ (∼90%) is notably higher than the largest value (66%) previously reported for a single CoFeB layer on PMN-30PT^1^.

**Figure 3:**
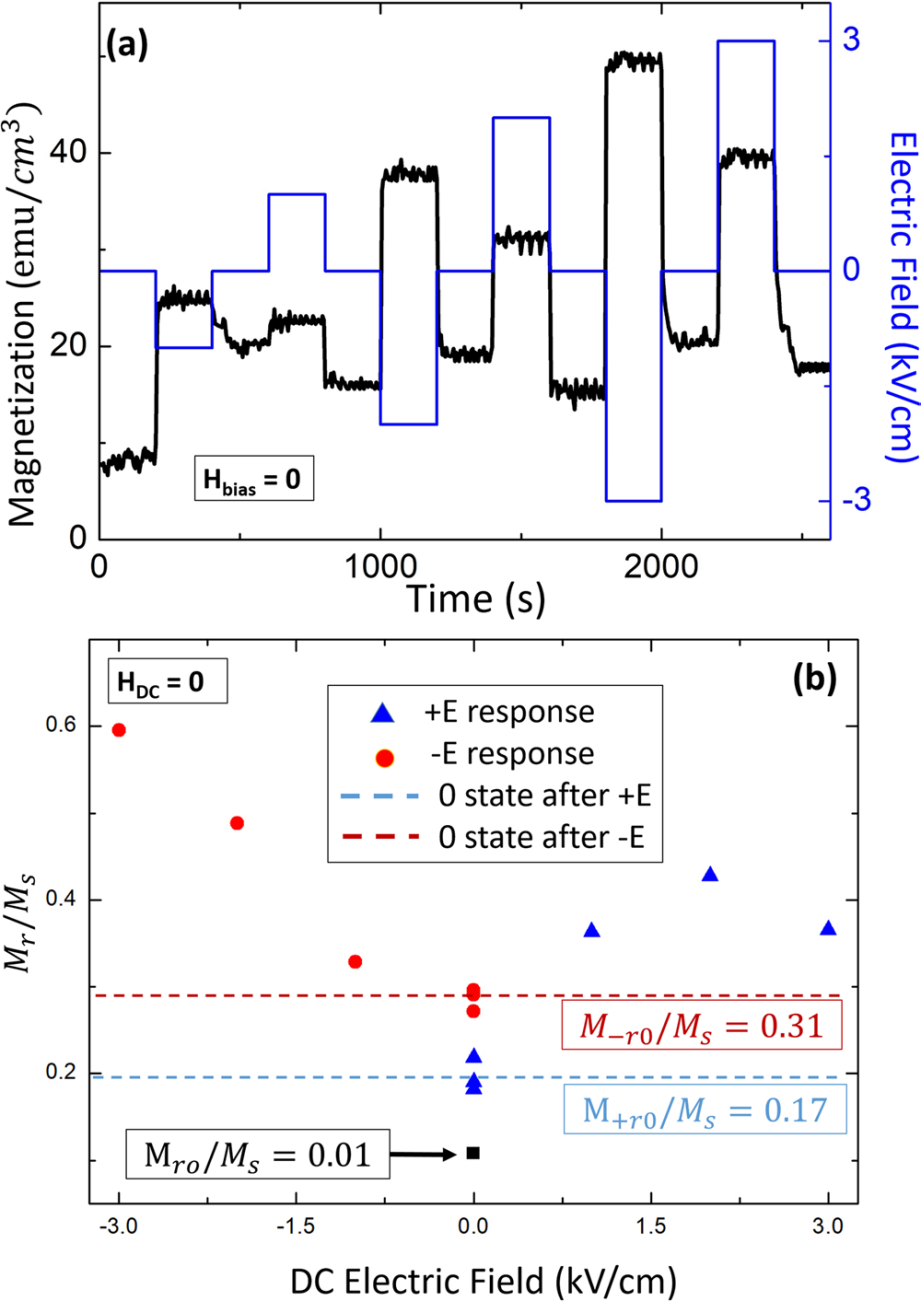
(**a**) Time vs. magnetization measurement under different magnetic fields. (**b**) Calculated ${M}_{r}/{M}_{s}$ ratios under different E_*DC*_ and *H*_*DC*_ = 0 Oe, data are shown for both increasing and decreasing E-field sweeps.

Figure 3(a) shows the induced magnetization response to changes in E_*DC*_ as a function of time taken along the OP direction while ${{\rm{H}}}_{DC}=0$. Data are given at various E_*DC*_ between 1 kV/cm and 3 kV/cm, beginning from a condition where the CFO nanopillars had been previously magnetized. In Fig. 3(a), six distinguishable states were induced under different positive (1, 2, 3 kV/cm) and negative (−1, −2, −3 kV/cm) E_*DC*_. The largest response was found for ${{\rm{E}}}_{DC}=-3kV/cm$, which corresponded to the point of maximum ${M}_{r}/{M}_{s}$ in Fig. 2(c). The magnetization direction with positive or negative E_*DC*_ did not switch, but increased in value following a trajectory corresponding to the M-H loop (see Fig. 2(a)). After removing E_*DC*_, two stable magnetization states were accessible depending on the E_*DC*_ direction.

These data in Fig. 3(a) reveal a strong coupling between the PMN-PT substrate and the BFO-CFO nanocomposite layer. The magnetic domains in the nanocomposite layer may rotate under ${{\rm{E}}}_{DC} < 3{\rm{kV}}/{\rm{cm}}$, resulting in good E_*DC*_ tunable properties. The converse magnetoelectric coefficient (*α*) was then calculated from the data in Fig. 3(a), using the equation: ${\rm{\alpha }}={\mu }_{0}\frac{{\rm{\Delta }}M}{{\rm{\Delta }}E}$, where ${\mu }_{0}$ is the permeability of free space, and α is in units of s/m. The value of α was estimated to be 1.3 × 10^−7^ *s*/*m*, again taken under ${{\rm{H}}}_{DC}=0$ in a previously magnetized state. This magnetization coupling coefficient is much higher than that previously reported (about 10^−10^ s/m) for BiFeO_3_-CoFe_2_O_4_/SrRuO_3_/SrTiO_3_ heterostructures^21^, and is close to values reported by Eerenstein *et al*.^35^ for LSMO/PMN-PT (2 × 10^−7^ s/m). However, this prior investigation^35^ found such high values only over a limited temperature range. Our results show a large magnetoelectric coupling tunable under E_DC_, which could be used over a wide range of temperatures below 375 K.

Two different magnetization values were found to be stable upon removing E_*DC*_, whose values were dependent on E_*DC*_ histories (see Fig. 3(a)). To better illustrate these two states, the value of the ${M}_{r}/{M}_{s}$ ratio is shown as a function of E_*DC*_ in Fig. 3(b). We note that these data were taken under ${{\rm{H}}}_{DC}=0$, beginning form a condition previously magnetized to M_*r*_. The two different stable values of the ${M}_{r}/{M}_{s}$ ratio found on removal of E_*DC*_ were ${M}_{-r0}/{M}_{s}=0.31$ (after negative E_*DC*_) and ${M}_{+r0}/{M}_{s}=0.17$ (after positive E_*DC*_), as illustrated by dashed lines in Fig. 3(b). After subsequent application of different E_*DC*_ (between 1 and 3 kV/cm), these values did not change. The ${M}_{r}/{M}_{s}$ ratio between ${M}_{-r0}$ and ${M}_{+r0}$ was 0.14. Previously in Fig. 2(c), the difference in the ${M}_{r}/{M}_{s}$ ratio value was shown to be ${M}_{-r0}/{M}_{s}-{M}_{+r0}/{M}_{s}=$0.46 − 0.35 = 0.11, which is close to the value of 0.14. The trends were also consistent with the data in Fig. 2(d), where it can be seen upon removing E_*DC*_ that the E-induced strain relaxed, but its value under E_*DC*_ = 0 was different between positive and negative bias sweeps. The highest ${M}_{r}/{M}_{s}$ ratio value was found to be ~0.60 at ${{\rm{E}}}_{DC}=-3\,kV/cm$. The original state (${M}_{0}$) is slightly larger than zero, which was due to equipment measurement error. These two different stable magnetization states accessible by E_*DC*_ offer a unique multi-state magnetization for logic and memory devices. The semi-volatile nature of the magnetostriction of the CFO nanopillars is due to changes in the phase stability of the PMN-PT substrate.

**Figure 4:**
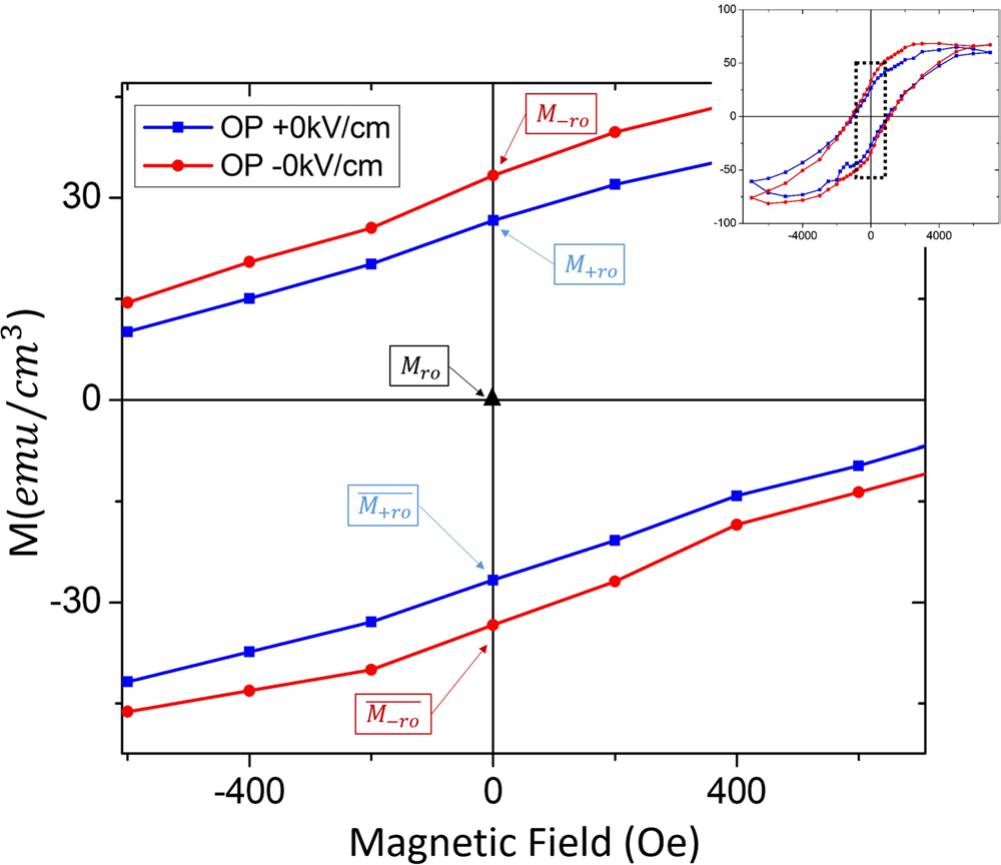
Enlarged M-H loop, showing magnetization states that accessible by different fields histories. The enlarged portion is shown in the dashed square area in the insert at upper right corner.

These ${M}_{r}/{M}_{s}$ ratio data clearly reveal three different states before and after E_*DC*_ (under H_*DC*_ = 0): ${M}_{r0}$, ${M}_{+r0}$ and ${M}_{-r0}$, as shown in Fig. 4. These states were for a previously magnetized condition, beginning from ${M}_{r0}$. As also shown in Fig. 4, the dual combination of ${{\rm{H}}}_{AC}$ (or ${{\rm{H}}}_{DC}$) followed by E_*DC*_ allow access to two additional oppositely magnetized states: $\overline{{M}_{+r0}}$ and $\overline{{M}_{-r0}}$. Thus, four or more (${\rm{N}}\ge 4$) stable remnant magnetization states can be accessed by E_*DC*_ in addition to ${M}_{r0}$. Previous studies of single phase magnetostrictive thin films on PMN-PT, such as CoNi/PMN-32PT^36^, have shown only two stable magnetization states; although, one study of FeAl/PIN-PMN-PT heterostructures^6^ reported four different states on removal of E_*DC*_ and ${{\rm{H}}}_{DC}$ that were accessed by ${{\rm{E}}}_{DC} > 4kV/cm$. We note that our investigations were done in a vertically integrated two phase ME layer on PMN-PT for $E < 3kV/cm$. This vertically integrated heterostructure with multistate ($N\ge 4$) values was tunable by E_*DC*_ are more relevant to integrated memories and logic than layer-by-layer ones. They have a high number of magnetic nanopillars; offer multiple stable magnetization states, which are accessible by E_*DC*_; and consume little power on changing states (i.e., passive). These multi-state heterostructures thus have the potential for neuromorphic-like applications.

There are several approaches to further improve the E-field induced coupling and to increase the number of stable magnetization states. One is the composition of the PMN-xPT substrate and its proximity to the morphotropic phase boundary (MPB). Our substrates were in the T-phase field with x = 38. However, compositions closer to the MPB (x = 35) have monoclinic (M), tetragonal (T), rhombohedral (R) and orthorhombic (O) phases that are close in energy^37^. In this case, application of E_*DC*_ results in induced phase transformations, where the induced phase remains metastable on removal of E_*DC*_; for example, the R → O phase transformation in PMN-32PT^38^. The availability of these metastable phases near the MPB offers the possibility of additional multistate magnetization values with E_*DC*_ for BFO-CFO/PMN-PT heterostructures. Second is the composition of the two phase BFO-CFO target, which was selected at 65at%BFO/35at%CFO in this study. By changing the composition of the BFO-CFO target, the aspect ratio of the CFO nanopillars could be modified, which would result in a change in the shape anisotropy (${K}_{shape}$). A previous report^15^ has shown with increasing aspect ratio of the CFO nanopillars (from 3:1 to 5:1) that the values of ${M}_{r}$ and ${H}_{C}$ along OP were significantly increased. Increasing ${K}_{shape}$ could also help stabilize additional non-volatile multi-state magnetization values.

## Conclusion

In summary, self-assembled nanopillar BFO-CFO two-phase layers have been deposited on SRO buffered PMN-xPT (100) substrates. Epitaxial growth of the vertical two-phase layers was shown by XRD, and a dense nanopillar surface was observed in AFM/MFM images. Large magnetization changes under applied E_*DC*_ were found along the easy magnetization axis, where the ${M}_{r}/{M}_{s}$ ratio exhibited a butterfly loop with E_*DC*_. The value of ${\rm{\Delta }}M/{{\rm{M}}}_{rDC}$ was calculated, and the maximum was found to be ∼90%. The converse magnetoelectric coupling coefficient was calculated to be 1.3×10^−7^ s/m. Real time changes in the magnetization with E_*DC*_ were measured, and multiple stable magnetization states ($N\ge 4$) were found on the removal of field.

## Methods

A 65%BFO-35%CFO composition ratio was chosen for the substrates. All thin films were deposited by PLD. PMN-38PT (100) single crystal substrates were grown by the Shanghai Institute of Ceramics Chinese Academy Sciences. Prior to the deposition, the substrates were cleaned with acetone and alcohol via ultrasonication. First, a 10 nm SRO layer was deposited on the PMN-38PT at 700 °C, 1.5 ${\rm{J}}/c{m}^{2}$ energy density and 150 mTorr ${O}_{2}$ atmosphere. After annealing under 700 °C and 150 mTorr ${O}_{2}$ atmosphere for 30 min, a 200 nm BFO-CFO heterostructure was deposited at 650 °C, 1.2 ${\rm{J}}/c{m}^{2}$ energy density and 90 mTorr ${O}_{2}$ atmosphere. The sample was then annealed at 700 °C and 100 Torr ${O}_{2}$. Crystal structures were determined by X-ray diffraction (Philips X’Pert system) scans. Magnetic hysteresis curves were recorded using a vibrating sample magnetometer (VSM, Lakeshore 7300 series). Atomic force microscopy (AFM) and magnetic force microscopy (MFM) images were obtained (Dimension 3100, Vecco), which were used to study the film surface quality and magnetic domain structures.
